# The entero-endocrine response following a mixed-meal tolerance test with a non-nutritive pre-load in participants with pre-diabetes and type 2 diabetes: A crossover randomized controlled trial proof of concept study

**DOI:** 10.1371/journal.pone.0290261

**Published:** 2023-08-25

**Authors:** Mirthe Muilwijk, Joline W. J. Beulens, Lenka Groeneveld, Femke Rutters, Marieke T. Blom, Valeria Agamennone, Tim van den Broek, Bart J. F. Keijser, Femke Hoevenaars

**Affiliations:** 1 Epidemiology and Data Science, Amsterdam UMC Location Vrije Universiteit Amsterdam, Amsterdam, Netherlands; 2 Amsterdam Public Health, Health Behaviours & Cardiovascular Diseases, Amsterdam Cardiovascular Sciences, Diabetes & Metabolism, Amsterdam, The Netherlands; 3 Department of General Practice, Amsterdam UMC Location Vrije Universiteit Amsterdam, Amsterdam, Netherlands; 4 Department of Microbiology & Systems Biology, TNO, Leiden, The Netherlands; 5 Department of Preventive Dentistry, Academic Centre for Dentistry Amsterdam (ACTA), Vrije Universiteit Amsterdam and University of Amsterdam, Amsterdam, The Netherlands; Karolinska Institutet, SWEDEN

## Abstract

**Introduction:**

This crossover randomized controlled trial (RCT) investigated differences in short-term entero-endocrine response to a mixed-meal tolerance test preceded by nutrient sensing between participants with pre-diabetes (pre-T2D) and type 2 diabetes (T2D). Additionally, differences in gut and oral microbiome composition between participants with a high and low entero-endocrine response were investigated.

**Research design and methods:**

Ten participants with pre-T2D and ten with T2D underwent three test days with pre-loads consisting of either swallowing water (control), or rinsing with a non-nutritive sweetener solution, or swallowing the sweetener solution before a mixed-meal tolerance test. Blood glucose-dependent insulinotropic polypeptide (GIP), glucagon-like peptide-1 (GLP-1), glucagon, glucose, insulin and peptide YY (PYY) were determined at t = -20, 0, 15, 30, 60, 120 and 240 minutes. The composition of the oral and gut microbiome at baseline were also determined.

**Results:**

The entero-endocrine response differed by pre-loads, e.g. a lower PYY response after swallowing the non-nutritive sweetener (-3585.2pg/mL [95% CI: -6440.6; -729.8]; p = 0.01). But it also differed by T2D status, e.g. a higher glucose, glucagon and PYY response was found in participants with T2D, compared to those with pre-T2D. Evidence for associations between the oral and gut microbiome composition and the entero-endocrine response was limited. Still, the level of entero-endocrine response was associated with several oral microbiome measures. Higher oral anterior α-diversity was associated with a lower PYY response (e.g. Inverse Simpson index -1357pg/mL [95% CI -2378; -336; 1.24]), and higher oral posterior α-diversitywith a higher GIP response (e.g. Inverse Simpson index 6773pg/mL [95% CI 132; 13414]) in models adjusted for sex, age and T2D status.

**Conclusions:**

Non-nutritive pre-loads influence the entero-endocrine response to a mixed-meal, and this effect varies based on (pre-)T2D status. The entero-endocrine response is likely not associated with the gut microbiome, and there is limited evidence for association with the α-diversity of the oral microbiome composition.

**Trial registration:**

**Trial register:** Netherlands Trial Register NTR7212, accessible through International Clinical Trials Registry Platform: ICTRP Search Portal (who.int).

## Introduction

Important risk factors for type 2 diabetes (T2D) include obesity, lack of physical activity, and energy-dense diets [1]. In keeping a balance between food intake and energy expenditure there is an important role for nutrient sensing and the production and release of hormones (entero-endocrine response) in response to meal intake [2]. Nutrient sensing takes place in the mouth and gut via taste receptors, and it is linked to neuronal signaling and metabolic responses, such as glucose homeostasis and satiety hormone release [3]. Food acceptance and choices may be affected by nutrient sensing as it influences appetite, appreciation and rewarding of food [4]. Moreover, nutrient sensing activates negative feedback loops to inhibit food intake [5]. By such feedback loops, non-nutritive sweeteners could reduce caloric intake while maintaining the contentment linked to taste [6].

Both glucose and non-nutritive sweeteners may activate receptors in the taste cells leading to a metabolic response, e.g. the release of glucagon-like peptide-1 (GLP-1) [7]. Human studies investigating the entero-endocrine response following intake of non-nutritive sweeteners have reported conflicting results [8]. Generally, postprandial glucose levels were not affected, with some studies reporting lower glycaemic responses. GLP-1 release was not altered in studies with an acute non-nutritive sweetener intake. However, when a non-nutritive pre-load was consumed some time prior to a glucose load, studies showed altered GLP-1 release [8]. For example, a study by Temizkan *et al*. reported an increase in GLP-1 after an oral glucose tolerance test (OGTT) when this was preceded by a sucralose pre-load. Interestingly, this effect was only observed in participants without T2D, while no such effect was observed in participants with T2D [9]. This suggests that diabetes status can influence entero-endocrine responses to sweeteners and glucose loads.

The non-nutritive sweetener stevia (steviol glycoside containing extracts) could be of special interest to people with T2D and potentially to prevent T2D, as stevia has been acknowledged to have glucose-lowering effects [10]. Moreover, it was demonstrated that stevia can reduce postprandial blood glucose levels in people with T2D [10, 11]. In the oral cavity and small intestine, stevia is able to interact with taste receptors. The activation of transient receptor potential cation channel subfamily M member 5 (TRPM5) by steviol glycosides potentiates glucose-induced insulin secretion, thus resembling the action of GLP-1 [12].

The gut and oral microbiome may play an important role in the various aspects of nutrient sensing [13], and both oral and gut microbiota were shown to differ across states of glycaemia [14–16]. Oral microbiota may for instance impact taste perception through various mechanisms. Examples thereof are the modulation and enhancement of the concentrations of tastants near taste receptors by the oral microbiome [17, 18], and by catabolization of salivary proteins [19]. Gut microbiota, on the other hand, may impact nutrient sensing through the detection of microbial fermentation products [20] via the microbiota-gut-brain axis [21] or by upregulating the expression of nutrient-responsive receptors and transporters [22]. Since nutrient sensing is important in the entero-endocrine response, both the oral and the gut microbiome may impact the entero-endocrine response.

Stevia could serve as an alternative to sucrose, but the entero-endocrine response to stevia remains unknown. Moreover, the influence of the gut and oral microbiome on the entero-endocrine response has not been investigated yet. Therefore, as a proof of concept, our crossover randomized controlled trial (RCT) set out to investigate the entero-endocrine response to a mixed-meal tolerance test performed 20 minutes after nutrient sensing in participants with pre-diabetes (pre-T2D) and T2D. A non-nutritive stevia sweetener was either used for rinsing or swallowed to compare the effect of nutrient sensing in the mouth and in the gut. The main objective was to investigate differences in response between participants with pre-T2D and those with T2D. Next, associations between gut and oral microbiome and the entero-endocrine response were assessed after the swallowed pre-load, thus considering both oral and gut sensing of the pre-load.

## Material and methods

### Study design

**Fig 1:**
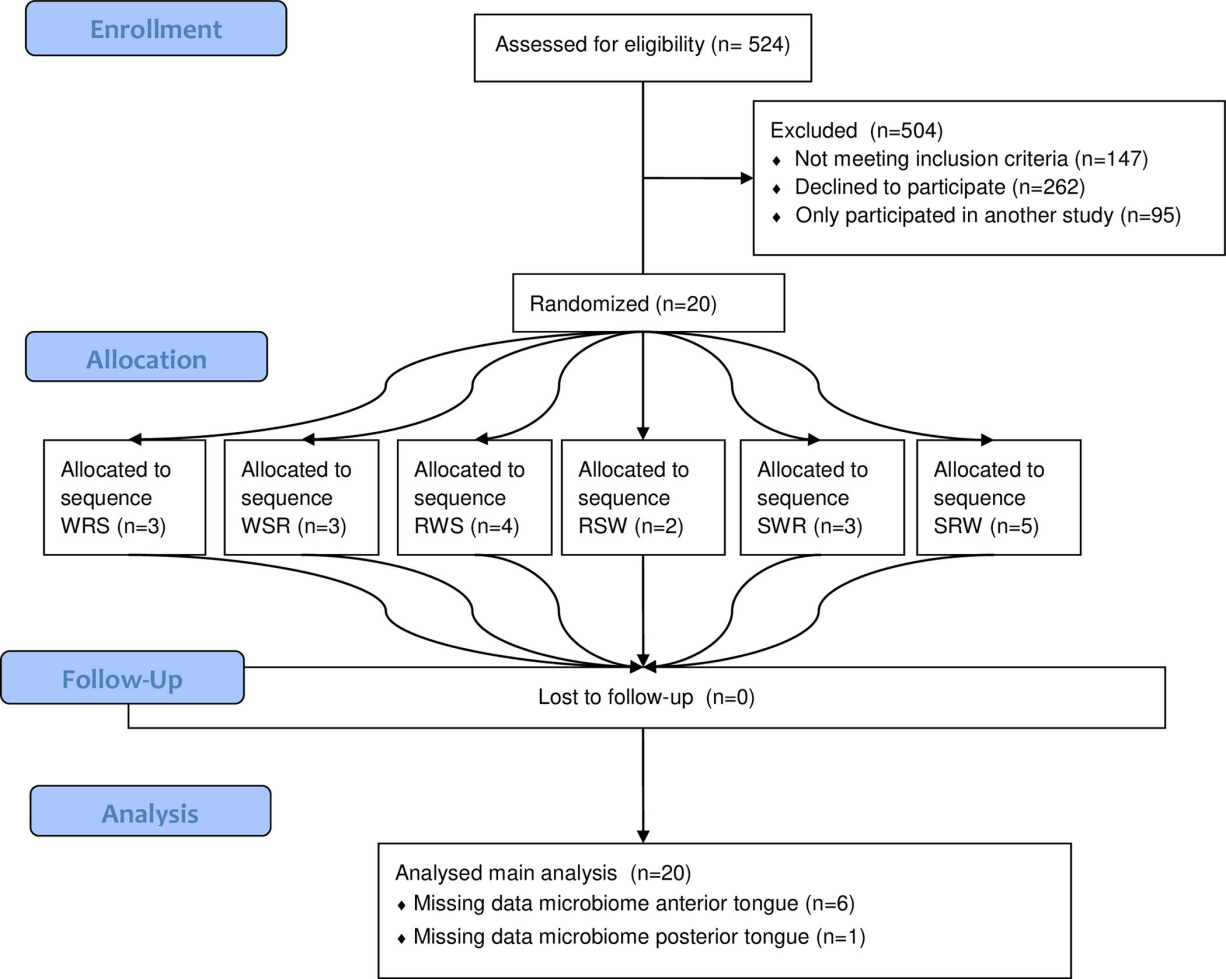
CONSORT flow diagram. Flow diagram showing the enrollment, allocation and follow–up of participants and finally data analyzed. W = Water rinsed, R = non–nutritive sweetener rinsed, S = non–nutritive sweetener swallowed.

**Fig 2:**
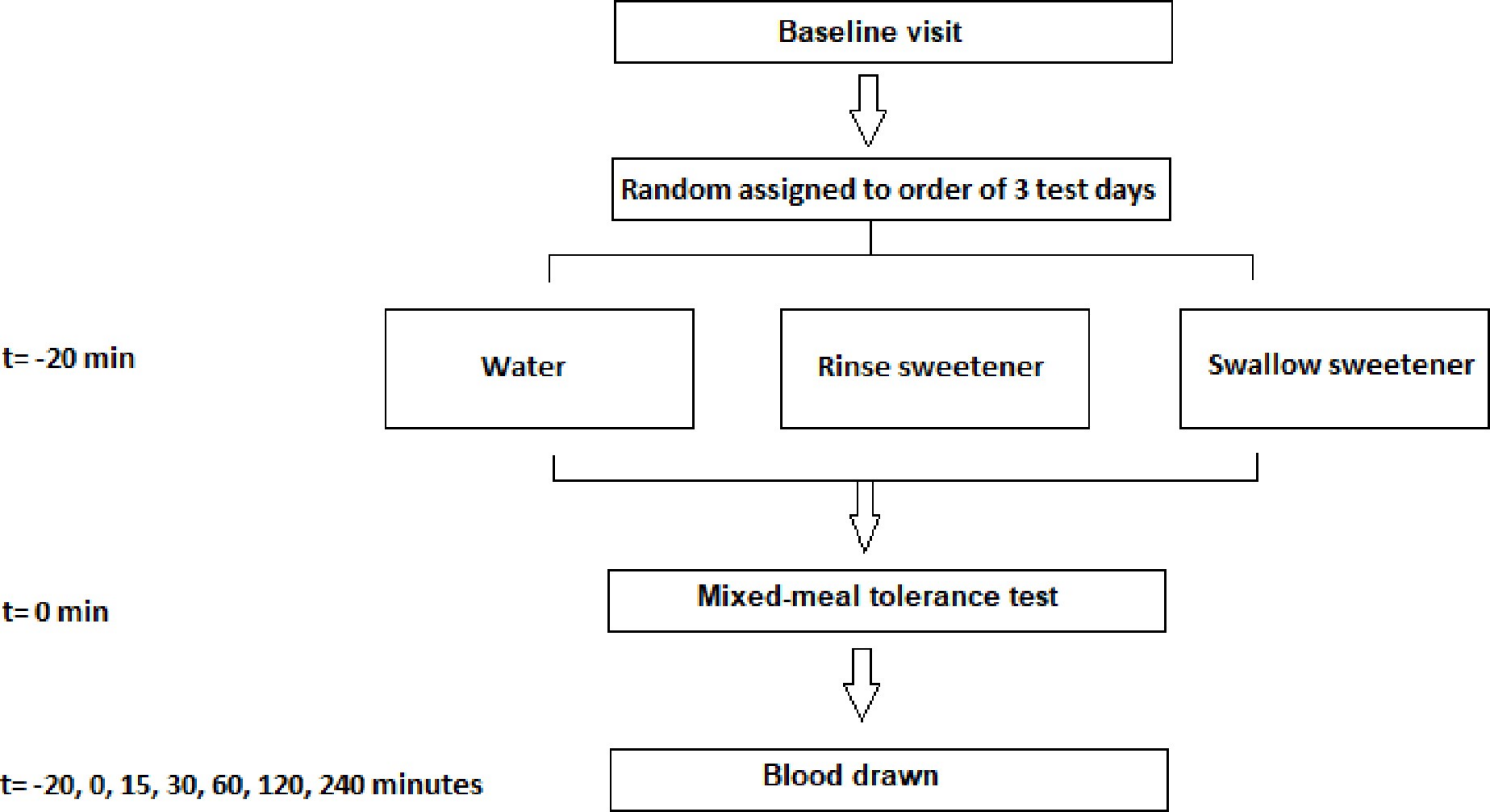
Procedure during test days. Participants came for a baseline visit and were then randomly assigned to an order of three test days. During each of the test days, participants received one of the three preloads 20 minutes before the mixed–meal tolerance test. Blood was drawn at t = –20, 0, 15, 30, 60, 120 and 240 minutes after the mixed–meal tolerance test.

In this crossover RCT, twenty participants with either pre-T2D or T2D received a mixed-meal tolerance test on three separate test days (Fig 1). During each of the test days, 20 minutes before the mixed-meal tolerance test, the participants were administered a pre-load of either water to swallow, a sweetener to rinse, or a sweetener to swallow (Fig 2). The order in which the three test procedures were administered to each participant was defined using a computerized random number generator. Blood was drawn immediately before the pre-load, before consumption of the mixed-meal tolerance test, and 15, 30, 60, 120 and 240 minutes after this drink, and was used to measure the entero-endocrine response.

Participants visited the Research Centre in Hoorn four times: once for a baseline visit, and three times for the study visits, with at least one week separating each visit to allow enough time for wash-out. Participants were asked to collect fecal samples at home prior to each visit, to determine the microbiome composition. Participants had to remain fasted from 22:00 the night before the test, with some water allowed, and were asked to avoid physical exercise and eating spicy foods during the 24 hours preceding the test. This study was conducted according to the principles of the Declaration of Helsinki. The study protocol was approved by the Medical Ethical Committee from VUmc, registration number NL63702.029.17 (S1 File). The trial was registered before commence of the study at the Netherlands Trial Register NTR7212, and can be accessed through the International Clinical Trials Registry Platform (ICTRP): ICTRP Search Portal (who.int) All participants provided written informed consent. The manuscript was written in line with the CONSORT guidelines (S2 File).

### Study population

A total of 524 people who originally participated in the Dutch arm of the DIRECT study were assessed for eligibility (Fig 2). DIRECT is a multicenter prospective cohort study to discover and validate biomarkers of glycaemic deterioration before and after the onset of T2D. Details of the DIRECT study have been described elsewhere [23]. Our inclusion criteria were being able to speak, write and understand Dutch. Exclusion criteria were: any significant medical reason for exclusion as determined by the investigator; having a history of medical or surgical (gastrointestinal) events that may significantly affect the study outcome; smoking; other medication for diabetes than oral medication (i.e. insulin); use of antibiotic medication in the last three months; alcohol consumption >21 units/week; and not agreeing to blood sampling during the study. From the initial 524 people, 147 did not meet the eligibility criteria (133 due to smoking). The remaining 377 people received written participant information and a phone call, after which a total of 115 people remained interested in the study. Finally, we selected ten participants with pre-T2D and ten with T2D, based on how well they could be matched on age, sex and BMI. This sample size is in line with previous studies that have been successful in demonstrating compositional differences in microbiome between groups of people with and without T2D [16, 24–26]. In both groups five men and five women were included. Mean age of the pre-T2D and T2D participants was 69.7 (SD 4.1) and 69.1 years (SD 3.7), while mean BMI was 29.2 (SD 2.5) and 29.5 kg/m^2^ (SD 2.4). All participants in the T2D group used oral medication for their T2D. The DIRECT study defined pre-T2D and T2D based on the American Diabetes Association 2011 criteria [27], and we verified the diabetes status by checking blood-glucose levels with a finger-prick (T2D if glucose >8.1mmol/L).

### Baseline visit: Anthropometric measures and tongue microbiota sampling

Anthropometric measurements were conducted during the baseline visit. Weight and height were measured while participants were barefoot and wearing light clothes. Body mass index (BMI) was calculated by dividing kilograms of weight by the square of height in meters. Waist circumference was measured midway between the lowest rib margin and the iliac crest.

To determine the composition of the tongue microbiota, anterior and posterior tongue swab samples were collected separately, as an ecological gradient in community composition is known to exist along the anterior-posterior axis of the tongue, resulting in two distinct niches [28]. Tongue anterior (TA) samples were collected by applying two strokes over the most anterior part of the tongue dorsum using a sterile micro brush. Tongue posterior (TP) samples were collected in a similar fashion, covering the posterior side of the tongue. Tongue swab samples were subsequently transferred to Eppendorf vials containing 250 μl RNAProtect solution and stored at -80 ⁰C until sequencing.

### Test day procedures

During each of the three test days, participants received a mixed-meal PhenFlex challenge tolerance test [29] consisting of a drink of 400 mL containing 75 g glucose, 20 g protein, 60 g palm olein, 0.5 g vanilla aroma, and 320 ml water (total 950 kCal). Before consuming the drink, participants received a 100 mL preload, which was meant to achieve either oral priming (i.e. activation of the sweet taste receptors in the mouth), or oral and small intestinal priming (activation of receptors in the mouth and small intestine), or no priming. Participants were allowed to drink the pre-loads in 20ml servings, and to rinse each serving for 15 seconds. On each of the three test days different preloads were administered. These were: (i) rinsing the mouth with a sweetener (referred to as ‘rinse’), to achieve oral priming; ii) swallowing the sweetener (referred to as ‘swallow’), to achieve small intestinal priming; and iii) water, as a control, to achieve no activation. The sweetener used was a steviol glycoside (Greensweet Stevia), which was administered as a solution of 0.12ml/100ml. This concentration simulated the sweetness of a soft drink such as Coca Cola (26gr sugar/250ml). The preload was offered to the participants 20 minutes before the mixed meal tolerance test drink [11, 30]. Blood was drawn immediately before the preload (t = -20), before consumption of the mixed-meal tolerance test drink (t = 0) and five times during the test (t = 15, 30, 60, 120 and 240 minutes). Samples were analyzed for glucose, insulin, glucose-dependent insulinotropic polypeptide (GIP), active GLP-1 and peptide YY (PYY).

### Fecal collection

Fecal samples were collected by the individual participants at home using a Fe-Col collection device (Alpha laboratories) and the OMNIgene GUT (DNA Genotek) collection kit. Samples were taken to the test centre by the participant on their test day, and then stored at -80⁰C until further processing. The sample collected at baseline was considered for the current analyses.

### Microbiome analyses: DNA isolation and amplicon sequencing

Microbiota from the tongue and faeces were characterized by sequence analysis of the V4 hypervariable region of the 16S ribosomal gene. The V4 hypervariable region provides profiles that adequately represent diverse communicates at the genus level, we aimed to obtain an overview of the microbial composition of the tongue, without necessarily achieving differentiation at the species level [31, 32]. DNA was extracted from the samples using the Qiagen Qiacube extraction protocol. To determine the amount of bacterial DNA, a quantitative polymerase chain reaction (qPCR) was performed using primers 16Suni-I-F CGA AAG CGT GGG GAG CAA A, 16Suni-I-R GTT CGT ACT CCC CAG GCG G, and 6-FAM MGB probe ATT AGA TAC CCT GGT AGT CCA that are specific for the bacterial 16S rRNA gene using RT PCR master mix (Diagenode, Seraing, Belgium) on an Applied Biosystems 7500 RT PCR system. For 16S rDNA amplicon sequencing of the V4 hypervariable region, 1 ng of DNA was amplified as described by Kozich *et al*. [33] with the exception that 33 cycles were used instead of 35, using F515/R806 primers [34]. A number of controls were included: mock samples comprising a mixture of 24 pure culture isolates, blanks, and pooled fecal and tongue samples. Primers included the Illumina adapters and a unique 8-nt sample index sequence key [33]. The amount of DNA per sample was quantified using the Quant-iT™ PicoGreen® dsDNA Assay Kit (Thermo Fisher Scientific). The amplicon libraries were pooled in equimolar amounts and purified using the IllustraTM GFXTM PCR DNA and Gel Band Purification Kit (GE Healthcare, Eindhoven, The Netherlands). Amplicon quality and size was analyzed on the Fragment Analyzer (Advanced Analytical). Paired-end sequencing of amplicons was conducted in five separate runs on the Illumina MiSeq platform (Illumina, Eindhoven, The Netherlands). Sequence pre-processing, analysis and classification was performed using modules implemented in the Mothur software platform v.1.31.2 [35]. Negative and positive controls were added for quality control [36, 37]. Sequences were grouped in operational taxonomic units by Minimal Entropy Decomposition (MED) using a minimum substantive abundance value (−M) of 500 [38]. Taxonomy was then assigned by querying the representative sequence of each oligotype against the SILVA database (release 132) [39].

### Laboratory analysis

Serum glucose was quantified using the hexokinase method (Instruchemie, Delfzijl, The Netherlands). Plasma levels of Insulin, GLP1 (active), glucagon and PYY (total) were measured using Meso Scale Discovery Diabetes combo kit 2, according to the manufacturer’s instructions.

### Data analysis

Baseline characteristics of participants are shown as means and standard deviations (SD), medians and interquartile ranges or frequency and percentages. Group differences were assessed by t-tests, Wilcoxon-signed rank tests and chi-square tests, respectively. All participants underwent the three test days, and data from all participants were included in the analysis. Two observations from one participant were missing (GLP1 at -20 and 0 minutes). Linear mixed effects models were used, which can handle missing time points. None of the co-variates was missing. The entero-endocrine response was characterized both as the total response by area under the curve (AUC) analyses, and by the response at different sampling time points. The AUC was calculated with the trapezoidal rule. First, we assessed effect modification by sex and T2D status. We stratified the analyses by T2D status, because we identified effect modification by T2D status for all metabolites (e.g. for insulin and GIP after the swallowed pre-load; p<0.001). We did not identify effect modification by sex (p>0.05), and did thus not stratify by sex. Next, sampling time points were tested as being independent to identify the time points at which the entero-endocrine response differed most. To investigate the association of nutrient sensing with endocrine responses, a mixed model for repeated measures with pre-treatment as exposure and the endocrine response as outcome was used, participants were included as a random effect.

Finally, associations between gut and oral microbiome and the entero-endocrine response (as continuous variable) were assessed after the swallowed pre-load, thus considering both oral and gut sensing of the pre-load. Microbiome data was rescaled and transformed using Wisconsin double and square root transformations. Composition for all samples was plotted using stacked bar charts in order to assess their composition in case a sample appeared anomalous. First, we assessed with multiple linear regression whether alpha diversity was associated with the entero-endocrine response, using the Inverse Simpson and Shannon diversity indices. Second, Canonical Correspondence Analysis (CCA) was applied to determine whether there was an association between the oral and fecal microbiome composition and the metabolic response. Participants with missing microbiome data (n = 6, anterior tongue; n = 1 posterior tongue) were omitted from these analyses as tongue swab samples were not missing at random. All microbiome analyses were conducted both unadjusted and adjusted for age, sex and T2D status.

All analyses were conducted in R studio version 4.0.3. The ‘lme4’ package was used for the generalized linear mixed models. We used the packages ‘vegan’ for nonmetric multidimensional scaling and PERMANOVA, ‘phyloseq’ for alpha-diversity, ‘CCA’ for RCCA and ‘ggplot2’ for visualization. A two-tailed p-value <0.05 was considered as statistically significant. We did not adjust for multiple-testing as this is a proof-of-concept study.

## Results

### Entero-endocrine response differs based on diabetes status

**Fig 3:**
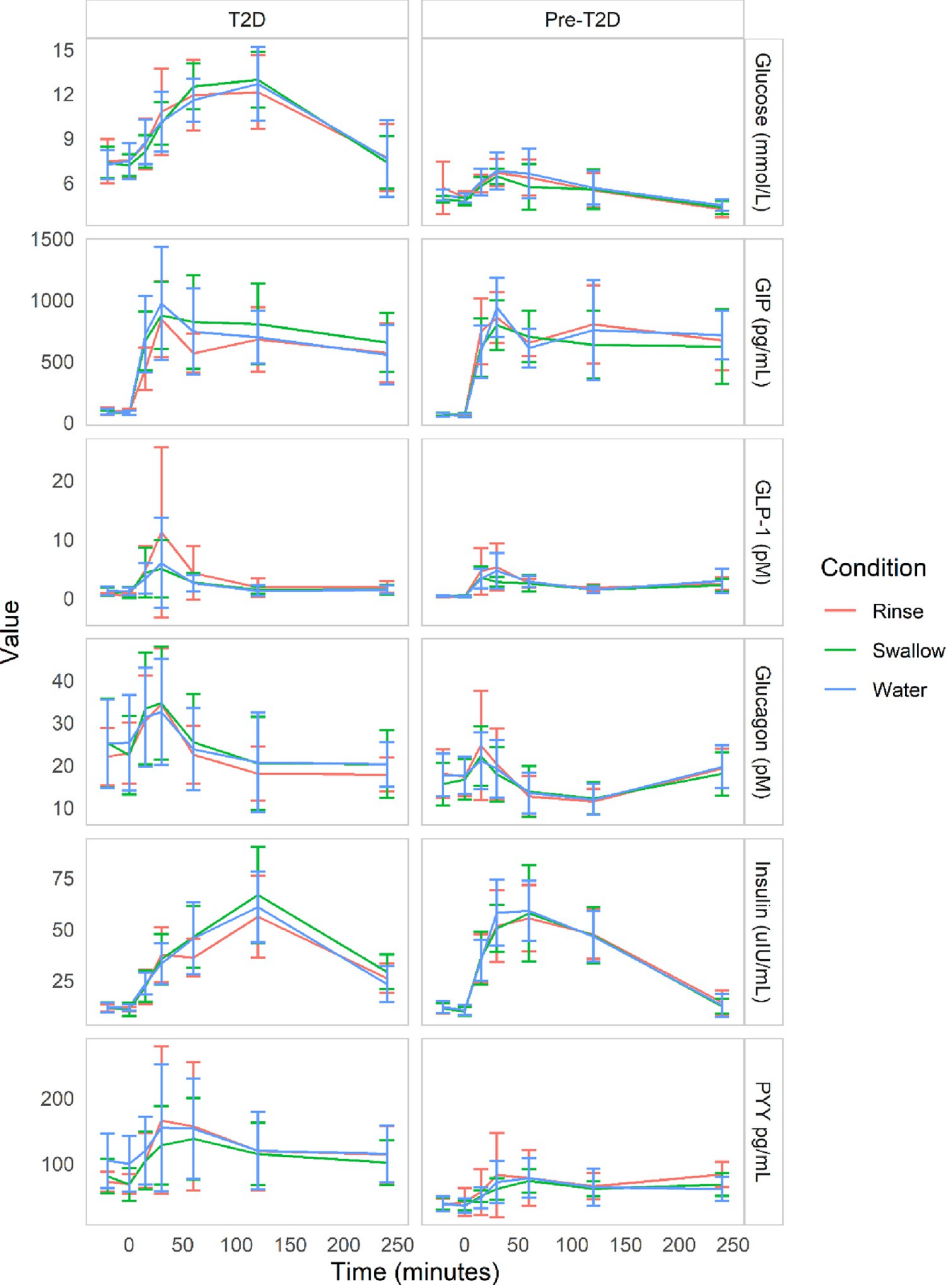
Entero–endocrine response following a mixed–meal tolerance test. Line plots of entero–endocrine response following a mixed–meal tolerance test, with different pre–loads 20 minutes preceding the mixed–meal, stratified by T2D status. The pre–loads were either swallowed water, a non–nutritive sweetener rinse, or swallowed non–nutritive sweetener. Means and standard deviations are shown for each of the time points and treatments.

Participants were well matched by T2D status, baseline characteristics are shown in Table 1. The entero-endocrine response to a mixed-meal test preceded by a non-nutritive stevia pre-load compared to a water pre-load differed between participants with T2D and those with pre-T2D Table 2. Blood glucose, glucagon, insulin, GIP, GLP-1 and PYY levels increased after the mixed-meal tolerance test in both participants with T2D and pre-T2D, regardless of the pre-load (Fig 3). The response in glucose, glucagon and PYY was higher among participants with T2D, compared to participants with pre-T2D, with either pre-load (e.g. p-value for the multiplicative interaction between diabetes status and the swallowed pre-load was 0.01 for glucose and <0.001 for insulin). The response in insulin after the swallow pre-load was higher, and the response in GIP after a rinse pre-load was lower in participants with T2D compared to those with pre-T2D. The GLP-1 response did not differ between participants with pre-T2D and T2D, with any of the pre-loads. All further analyses were stratified by diabetes status.

**Table 1:** Baseline characteristics of participants, stratified by T2D status.

	Participants with pre-T2D (n = 10)	Participants with T2D (n = 10)	P-value
**Mean age (years)**	69.7 (4.1)	69.1 (3.7)	0.77
**N female**	5 (50%)	5 (50%)	1.00
**Mean BMI (kg/m** ^**2**^ **)**	29.2 (2.5)	29.5 (2.4)	0.78
**Mean waist circumference (cm)**	105 (11.5)	104 (9.9)	0.89
**Mean systolic blood pressure**	133 (11.9)	141 (11.3)	0.15
**Mean diastolic blood pressure**	80.5 (5.3)	78.0 (4.2)	0.26
**Median glucose fingerprick (mmol/L)**	5.4 [5.1; 5.5]	6.9 [6.5; 8.7]	0.004
**Oral diabetes medication n**	0 (0%)	10 (100%)	<0.001

Data are mean (SD), median [IQR] or n(%). P–values from the corresponding t–tests, Wilcoxon–signed rank tests and chi–square tests, are shown.

**Table 2:** Entero–endocrine response (area under the curve; AUC) to a mixed–meal tolerance test preceded by a non–nutritive sweetener pre–load (either mouth rinse or swallowing), compared to water pre–load, stratified by T2D status. Mean and SD is shown for AUCs. β is the regression coefficient for the difference compared to water. 95% CI = 95% confidence interval.

	Water		Rinse				Swallow			
	AUC	β	AUC	β	95% CI	p-value	AUC	β	95% CI	p-value
**GIP** (pg/mL)										
Pre-T2D	168923 (61223)	1.0 (ref)	173340 (55583)	4417.0	-791.1; 9625.2	0.10	154153 (50572)	**-14769.4**	**-19977.6; -9561.2**	***<0*.*001***
T2D	163668 (74052)	1.0 (ref)	148061 (63918)	**-15607.2**	**-24833.9; -6380.6**	***0*.*001***	179942 (72174)	**16274.7**	**7048.0; 25501.4**	***<0*.*001***
**GLP-1** (pM)										
Pre-T2D	589 (264)	1.0 (ref)	607 (285)	17.4	-48.8; 83.7	0.61	496 (242)	**-93.4**	**-159.6; -27.1**	***0*.*006***
T2D	523 (504)	1.0 (ref)	808 (967)	**285.1**	**153.1; 417.0**	***<0*.*001***	530 (411)	6.7	-125.3; 138.7	0.92
**Glucagon** (pM)										
Pre-T2D	4079 (1512)	1.0 (ref)	4053 (1533)	-26.2	-216.9; 164.4	0.79	3963 (1628)	-116.0	-306.6; 74.6	0.24
T2D	6029 (3264)	1.0 (ref)	5540 (1533)	**-489.2**	**-787.2; -191.1**	***0*.*002***	6111 (3484)	81.2	-216.8; 379.2	0.59
**Glucose** (mmol/L)										
Pre-T2D	1453 (224)	1.0 (ref)	1412 (228)	-40.5	-83.1; 2.2	0.06	1375 (205)	**-77.7**	**-120.3; -35.0**	***<0*.*001***
T2D	2678 (630)	1.0 (ref)	2664 (701)	-14.4	-92.0; 63.3	0.72	2715 (452)	37.0	-40.7; 114.6	0.35
**Insulin** (μIU/mL)										
Pre-T2D	9717 (2296)	1.0 (ref)	9589 (2283)	-127.8	-556.8; 301.2	0.56	9504 (2283)	-212.2	-641.2; 216.8	0.33
T2D	10329 (3113)	1.0 (ref)	9716 (2283)	**-613.3**	**-910.1; -316.5**	***<0*.*001***	11258 (4052)	929.3	**632.5; 1226.0**	***<0*.*001***
**PYY** (pg/mL)										
Pre-T2D	16218 (7824)	1.0 (ref)	18135 (8378)	**1917.1**	**730.2; 3103.9**	***0*.*002***	16001 (4111)	-216.6	-1403.5; 970.2	0.72
T2D	32555 (18870)	1.0 (ref)	31798 (19140)	-756.8	-3612.2; 2098.6	0.60	28970 (14448)	-3585.2	**-6440.6; -729.8**	***0*.*01***

### Entero-endocrine response differs by pre-load

The increase in AUC entero-endocrine response of the rinse or swallow pre-load was compared to the water pre-load (Fig 3, Table 2). When comparing the rinse to the water pre-load, participants with pre-T2D had a significantly larger response in PYY (1917.1 pg/mL [95% CI 730.2; 3103.9]) after the rinse, while no differences were observed for glucose, glucagon, insulin, GIP or GLP-1. Among participants with T2D, the rinse pre-load compared to water elicited a statistically significant smaller response for glucagon, insulin and GIP, a larger response in GLP-1, and no statistically different response for glucose and PYY. When considering the swallow pre-load, in participants with pre-T2D the entero-endocrine response was generally smaller compared to the water pre-load, with statistically significant differences observed for glucose (-77.7 mmol/L [95% CI -120.3; -35.0]), GIP and GLP-1. In participants with T2D, the swallow pre-load resulted in significantly lower PYY response (-3585.2 pg/mL [95% CI -6440.6; -729.8]) compared to the water pre-load, while the response in insulin and GIP were significantly higher, and no significant difference in response was observed for glucose, glucagon or GLP-1. No statistically significant differences in entero-endocrine response following the mixed-meal test with different pre-loads were observed with the mixed-model analysis, considering different time-points.

### Oral microbiome diversity correlates with PYY and GIP response

First we assessed basic sequencing and data quality characteristics. The gut microbiome consisted of a total of 484 operational taxonomic units (OTUs), total abundances per sample ranged from 8939 to 10299 counts per sample. The oral microbiome consisted of a total of 110 OTUs, with abundances ranging from 8895 to 9735 counts per sample. Both α-diversity and β-diversity of the gut microbiome were not statistically significant associated with the entero-endocrine response Tables 3 and 4. For oral microbiome composition we observed statistically significant associations between oral anterior α-diversity and PYY, and posterior α-diversity and GIP. A higher oral posterior α-diversity was associated with a lower PYY response for both the Shannon and the Inverse Simpson index in the unadjusted models. After adjustment for sex, age and T2D status the association remained statistically significant for the Inverse Simpson index (-1357pg/mL [95% CI -2378; -336; 1.24]), but not for the Shannon index. A higher oral posterior α-diversity was associated with a higher GIP response for both the Shannon and Inverse Simpson index, and in both unadjusted and for sex, age and T2D status adjusted models (e.g. fully adjusted Inverse Simpson index 6773pg/mL [95% CI 132; 13414]). No statistically significant associations were observed between the α-diversity of the oral anterior microbiome and glucose, glucagon, insulin, GIP or GLP-1; not between α-diversity of the oral posterior microbiome and glucose, glucagon, insulin, GLP-1 or PYY; and finally, the β-diversity of the oral microbiome was not statistically significant associated with any of the entero-endocrine measures.

**Table 3:** Multiple linear regression of the association between alpha diversity and insulin response following a mixed–meal tolerance test following a mixed–meal test with a swallowed non–nutritive pre–load.

Microbiome	Entero-endocrine response	Coefficient	95% CI	p-value
**Gut**	**Glucose** (mmol/L)			
Shannon index	Unadjusted model	-21.8	-646; 602	0.94
Shannon index	+ sex, age, T2D status	92.6	-243; 428	0.56
Inverse Simpson	Unadjusted model	-2.25	-24.2; 19.7	0.83
Inverse Simpson	+ sex, age, T2D status	2.62	-10.1; 15.3	0.67
**Gut**	**Glucagon** (pM)			
Shannon index	Unadjusted model	751	-1620; 3122	0.51
Shannon index	+ sex, age, T2D status	905	-1622; 3431	0.46
Inverse Simpson	Unadjusted model	5.62	-79.0; 90.3	0.89
Inverse Simpson	+ sex, age, T2D status	9.49	-87.3; 106	0.84
**Gut**	**Insulin** (μIU/mL)			
Shannon index	Unadjusted model	-456	-3239; 2327	0.73
Shannon index	+ sex, age, T2D status	-589	-3712; 2535	0.69
Inverse Simpson	Unadjusted model	8.34	-90.1; 106.8	0.86
Inverse Simpson	+ sex, age, T2D status	9.51	-108.5; 127.6	0.87
**Gut**	**GIP** (pg/mL)			
Shannon index	Unadjusted model	16905	-34621; 68431	0.50
Shannon index	+ sex, age, T2D status	24944	-32441; 82328	0.37
Inverse Simpson	Unadjusted model	752	-1052; 2556	0.39
Inverse Simpson	+ sex, age, T2D status	1226	-889; 3341	0.24
**Gut**	**GLP-1** (pM)			
Shannon index	Unadjusted model	89.1	-852; 1213	0.50
Shannon index	+ sex, age, T2D status	-51.1	-279; 176.6	0.64
Inverse Simpson	Unadjusted model	1.72	-8.02; 11.5	0.72
Inverse Simpson	+ sex, age, T2D status	-6.10	-14.1; 1.86	0.12
**Gut**	**PYY** (pg/mL)			
Shannon index	Unadjusted model	1857	-22913; 54034	0.71
Shannon index	+ sex, age, T2D status	2193	-7831; 12217	0.65
Inverse Simpson	Unadjusted model	-74.2	-433; 285	0.67
Inverse Simpson	+ sex, age, T2D status	-109	-484; 266	0.55
**Oral anterior**	**Glucose** (mmol/L)			
Shannon index	Unadjusted model	-651	-1551; 250	0.14
Shannon index	+ sex, age, T2D status	-150	-806; 506	0.62
Inverse Simpson	Unadjusted model	-51.5	-149; 45.8	0.27
Inverse Simpson	+ sex, age, T2D status	-20.7	-83.3; 42.0	0.48
**Oral anterior**	**Glucagon** (pM)			
Shannon index	Unadjusted model	-3330	-7570; 911	0.11
Shannon index	+ sex, age, T2D status	-2446	-8018; 3126	0.35
Inverse Simpson	Unadjusted model	-389	-814; 36.7	0.07
Inverse Simpson	+ sex, age, T2D status	-315	-832; 203	0.20
**Oral anterior**	**Insulin** (μIU/mL)			
Shannon index	Unadjusted model	-30.5	-2955; 2894	0.98
Shannon index	+ sex, age, T2D status	667	-3275; 4608	0.71
Inverse Simpson	Unadjusted model	-13.7	-317; 290	0.92
Inverse Simpson	+ sex, age, T2D status	24.8	-360; 410	0.89
**Oral anterior**	**GIP** (pg/mL)			
Shannon index	Unadjusted model	-12344	-113657; 88970	0.80
Shannon index	+ sex, age, T2D status	-45658	-158720; 67404	0.38
Inverse Simpson	Unadjusted model	-1813	-12294; 8669	0.71
Inverse Simpson	+ sex, age, T2D status	-5084	-15890; 5722	0.31
**Oral anterior**	**GLP-1** (pM)			
Shannon index	Unadjusted model	-60.3	-440; 320	0.74
Shannon index	+ sex, age, T2D status	-297	-639; 45.7	0.08
Inverse Simpson	Unadjusted model	-15.8	-54.2; 22.6	0.39
Inverse Simpson	+ sex, age, T2D status	-31.4	-63.3; 0.32	0.05
**Oral anterior**	**PYY** (pg/mL)			
Shannon index	Unadjusted model	-12191	-22998; -1385	***0*.*03***
Shannon index	+ sex, age, T2D status	-11333	-23543; 877	0.07
Inverse Simpson	Unadjusted model	-1310	-2411; -209	***0*.*02***
Inverse Simpson	+ sex, age, T2D status	-1357	-2378; -336	***0*.*01***
**Oral posterior**	**Glucose** (mmol/L)			
Shannon index	Unadjusted model	-94.2	-1092; 904	0.85
Shannon index	+ sex, age, T2D status	242	-284; 767	0.34
Inverse Simpson	Unadjusted model	-19.4	-104; 65.5	0.64
Inverse Simpson	+ sex, age, T2D status	13.2	-33.0; 59.4	0.55
**Oral posterior**	**Glucagon** (pM)			
Shannon index	Unadjusted model	-755	-4602; 3092	0.68
Shannon index	+ sex, age, T2D status	215	-3770; 4199	0.91
Inverse Simpson	Unadjusted model	-79.4	-408; 249	0.62
Inverse Simpson	+ sex, age, T2D status	3.83	-339; 347	0.98
**Oral posterior**	**Insulin** (μIU/mL)			
Shannon index	Unadjusted model	370	-2198; 2937	0.76
Shannon index	+ sex, age, T2D status	708	-2324; 3740	0.62
Inverse Simpson	Unadjusted model	3.71	-216; 224	0.97
Inverse Simpson	+ sex, age, T2D status	31.22	-232; 294	0.80
**Oral posterior**	**GIP** (pg/mL)			
Shannon index	Unadjusted model	80099	9787; 150441	***0*.*03***
Shannon index	+ sex, age, T2D status	86687	12449; 160925	***0*.*03***
Inverse Simpson	Unadjusted model	6019	-225; 12263	0.06
Inverse Simpson	+ sex, age, T2D status	6773	132; 13414	***0*.*05***
**Oral posterior**	**GLP-1** (pM)			
Shannon index	Unadjusted model	234	-190; 659	0.26
Shannon index	+ sex, age, T2D status	63.7	-292; 419	0.71
Inverse Simpson	Unadjusted model	18.2	-18.4; 54.7	0.31
Inverse Simpson	+ sex, age, T2D status	2.92	-27.8; 33.7	0.84
**Oral posterior**	**PYY** (pg/mL)			
Shannon index	Unadjusted model	-579	-17090; 15933	0.94
Shannon index	+ sex, age, T2D status	283	-15924; 16489	0.97
Inverse Simpson	Unadjusted model	-308	-1711; 1096	0.65
Inverse Simpson	+ sex, age, T2D status	-221	-1611; 1169	0.74

**Table 4:** PERMANOVA results based on Bray–Curtis dissimilarities using abundance data for microbiome.

Response		ChiSquare	F	Pr (>F)
**Gut microbiome**				
**Glucose** (mmol/L)	Unadjusted model	0.04331	0.8150	0.68
	+ sex, age, T2D status	0.04331	0.8372	0.65
**Glucagon** (pM)	Unadjusted model	0.0584	1.1153	0.29
	+ sex, age, T2D status	0.0584	1.1464	0.25
**Insulin** (μIU/mL)	Unadjusted model	0.0379	0.7097	0.86
	+ sex, age, T2D status	0.0379	0.7163	0.84
**GIP** (pg/mL)	Unadjusted model	0.0375	0.7008	0.86
	+ sex, age, T2D status	0.0375	0.7158	0.84
**GLP-1** (pM)	Unadjusted model	0.0610	1.1688	0.22
	+ sex, age, T2D status	0.0610	1.1651	0.23
**PYY** (pg/mL)	Unadjusted model	0.0449	0.8460	0.59
	+ sex, age, T2D status	0.0449	0.8989	0.57
**Oral anterior microbiome**				
**Glucose** (mmol/L)	Unadjusted model	0.0889	1.1712	0.31
	+ sex, age, T2D status	0.0889	1.0209	0.55
**Glucagon** (pM)	Unadjusted model	0.1249	1.7132	0.16
	+ sex, age, T2D status	0.1249	1.5065	0.22
**Insulin** (μIU/mL)	Unadjusted model	0.1436	2.0125	0.09
	+ sex, age, T2D status	0.1436	1.9098	0.11
**GIP** (pg/mL)	Unadjusted model	0.0686	0.8843	0.46
	+ sex, age, T2D status	0.0686	0.8239	0.53
**GLP-1** (pM)	Unadjusted model	0.0743	0.9635	0.43
	+ sex, age, T2D status	0.0743	0.8139	0.56
**PYY** (pg/mL)	Unadjusted model	0.1035	1.3846	0.21
	+ sex, age, T2D status	0.1035	1.3219	0.24
**Oral posterior microbiome**				
**Glucose** (mmol/L)	Unadjusted model	0.0475	0.8475	0.56
	+ sex, age, T2D status	0.0475	0.8136	0.59
**Glucagon** (pM)	Unadjusted model	0.1466	1.0889	0.35
	+ sex, age, T2D status	0.0602	1.0156	0.42
**Insulin** (μIU/mL)	Unadjusted model	0.0300	0.5265	0.87
	+ sex, age, T2D status	0.0300	0.5029	0.89
**GIP** (pg/mL)	Unadjusted model	0.0625	1.1325	0.32
	+ sex, age, T2D status	0.0625	1.1044	0.35
**GLP-1** (pM)	Unadjusted model	0.0902	1.6859	0.10
	+ sex, age, T2D status	0.0902	1.5988	0.13
**PYY** (pg/mL)	Unadjusted model	0.0815	1.5078	0.12
	+ sex, age, T2D status	0.0815	1.4794	0.13

## Discussion

Our study suggests that non-nutritive stevia pre-loads influence the entero-endocrine response following a mixed-meal tolerance test, and that this effect is different in participants with and without T2D. This study is the first to explore associations between the microbiome and the entero-endocrine response following a mixed-meal tolerance test combined with a non-nutritive pre-load. We found limited evidence for associations between the α-diversity of the oral microbiome composition and the entero-endocrine response, and no evidence for associations between gut microbiome composition and the entero-endocrine response.

This study showed that downstream glucose response levels following a mixed-meal tolerance test were not affected by mouth rinsing with a non-nutritive sweetener pre-load. Among participants with pre-T2D, but not among those with T2D, glucose levels decreased after swallowing the non-nutritive pre-load. This is in line with other studies, which reported either glucose levels not to be affected or to be somewhat lower after a swallowed pre-load with a non-nutritive sweetener [8]. Similar to our study, Temizkan *et al*. showed that the glucose response differs between participants with and without T2D [9]. Combined, these findings suggest that nutrient sensing in the small intestine may lead to a reduction in glucose levels in people without T2D, but not in those with T2D. Where this suggests that stevia only interacted with taste receptors in the small intestine, opposite results were found for the glucagon response. Levels were lower after a rinsed (oral sensing) non-nutritive pre-load among participants with T2D, but not in those with pre-T2D. This suggests that stevia, indeed, interacts with taste receptors in the oral cavity as well, which is confirmed by the response observed for the other metabolites included in our study. In general, levels of the satiety hormone PYY increase following food intake. A study with an *ex vivo* porcine intestinal model showed that stevia may induce PYY release [40]. Our study showed mixed findings. PYY response increased after a rinsed (oral sensing) non-nutritive pre-load in participants with pre-T2D, but not in those with T2D. After a swallowed (additional small intestinal sensing) non-nutritive pre-load, PYY response decreased in participants with T2D, but not in those with pre-T2D. Our *in vivo* results in humans thus differ from earlier findings in the *ex vivo* porcine model. Future human studies are needed to confirm our findings.

GIP and GLP-1 increase after nutrient sensing, and stimulate secretion of insulin [41]. Margolskee *et al*. showed that artificial sweeteners can induce nutrient sensing in the gut and lead to the secretion of incretins GLP-1 and GIP [7]. Our study showed, in participants with T2D, a smaller GIP response and a larger GLP-1 response in the first 30 minutes following a mixed-meal tolerance test when this is preceded by a non-nutritive pre-load rinse compared to a water pre-load. The effect was opposite with swallowing (small intestinal nutrient sensing involved) the non-nutritive pre-load; GIP response was enhanced in those with T2D. However, GIP and GLP-1 responses were lower among participants with pre-T2D. Other studies also indicated that non-nutritive sweeteners may influence the GLP-1 response, although results are conflicting [8, 9]. In contrast to our study, Temizkan *et al*. e.g. reported an enhanced GLP-1 response among participants without T2D, but not in those with T2D [8, 9]. The conflicting results may be due to differences in study designs: for instance, the type of non-nutritive sweetener may influence nutrient sensing and the ensuing metabolic response. Our study used steviol glycoside as non-nutritive sweetener, while the study by Temizkan *et al*. used both aspartame and sucralose. That study already reported conflicting results for an aspartame compared to a sucralose non-nutritive pre-load with other study parameters kept identical [9]. In the oral cavity and small intestine steviol glycosides activate the TRPM5 receptor, which potentiates glucose-induced insulin secretion, resembling the action of GLP-1 [12]. However, we did not see an effect of the pre-load on insulin levels in participants with pre-T2D. Amongst those with T2D, we observed that the rinsed pre-load (oral nutrient sensing) lead to a decrease in insulin levels, and the swallowed (also small intestinal nutrient sensing) pre-load to an increase in insulin levels.

Our explorative study found no evidence for the gut microbiome composition to be associated with the entero-endocrine response. Other studies, however, suggest that the gut microbiome may impact the entero-endocrine response, for instance via production of short-chain fatty acids, which can activate G-protein-coupled receptors such as GPR41 and GPR43 expressed in colon epithelial cells where they stimulate the secretion of gut hormones such as GLP-1 and PYY [42]. We found limited evidence for associations between oral microbiome composition and the entero-endocrine response. In our study, α-diversity of the anterior oral microbiome was associated with the PYY response, while α-diversity of the posterior oral microbiome was associated with the GIP response. Studies in rodents suggest that the bacterially-derived lipopolysaccharide (LPS) may impact the response to sucrose, e.g. by downregulation of sweet taste receptor genes in taste buds [18].

Our study is not exempt from limitations. First, sample size was based on previous studies, since formal power calculations with regard to the gut and oral microbiome composition are difficult to perform due to variability and previous poor clinical trials [43]. Although we found indications that the oral microbiome composition is associated with the entero-endocrine response, the study could have benefitted from a larger sample size, for instance to better understand which specific microbiome species are most important. Larger studies may also consider adjusting for multiple-testing to avoid chance findings. Second, many different factors may influence the entero-endocrine response, including but not limited to the timing of a pre-load, the presence of other macronutrients and the type of non-nutritive sweetener. Moreover, although steviol glycosides are generally considered safe [44], we should consider that the metabolic activity of non-nutritive sweeteners should be thoroughly investigated to prevent adverse outcomes [45]. Third, our study only included Europeans, and results may differ for people of different ethnic background. Finally, future studies should consider that the use of non-nutritive sweeteners may impact the gut microbiome composition, which may then have either a detrimental or beneficial effect on T2D risk [46]. Despite these limitations our study was the first to show that a stevia non-nutritive pre-load influences the entero-endocrine response to a mixed-meal, and this effect varies based on diabetes status. Moreover, it was the first study to explore associations between the gut and oral microbiome composition and the entero-endocrine response following meal intake.

## Conclusions

Pre-loads with a non-nutritive sweetener can influence the entero-endocrine response to a mixed-meal tolerance test. Because steviol glycosides can influence the entero-endocrine response, they could potentially be used to develop dietary approaches based on inducing sweet-taste perception while reducing caloric intake. This might be of special interest to those with, or at high risk for T2D. In this study, we did not test whether changes in the entero-endocrine response were accompanied by changes in the satiety or behavior of participants. The response to a mixed-meal tolerance test with a stevia non-nutritive pre-load differs between participants with pre-T2D and T2D. Future studies and product development should thus consider that the effect of steviol glycosides on the entero-endocrine response may differ by population subgroups. Our study found limited evidence for an association of the oral microbiome the entero-endocrine response, and none for the gut microbiome, yet future well-powered studies may be able to investigate this further.

## Supporting information

S1 File Study protocol. (DOCX)

S2 File CONSORT 2010 checklist of information to include when reporting a randomised trial*. (DOC)
