# Supplementary material for: The entero-endocrine response following a mixed-meal tolerance test with a non-nutritive pre-load in participants with pre-diabetes and type 2 diabetes: A crossover randomized controlled trial proof of concept study
Source: PLoS One. 2023 Aug 25;18(8):e0290261. doi: 10.1371/journal.pone.0290261 (PMC10456129; doi:10.1371/journal.pone.0290261)
Supplement: S1 File — (DOCX) [file pone.0290261.s001.docx]

**Study protocol DIABOS**

**Rationale:**

Diabetes is a worldwide problem with a heavy burden on healthcare costs and it is associated with a variety of complications. Previous research has shown that people with type 2 diabetes may have altered mechanisms of nutrient sensing, which interferes with the response of the body via metabolic, endocrine and neural changes. Nutrient sensing starts in the mouth and is then continued in the gut by taste perception via receptors. The detection of sweet taste determines metabolic responses such as glucose homeostasis and satiety hormone release. Alterations of these functions can contribute to the onset of diabetes and obesity. The oral and gut microbiota interacts with host metabolism and physiology in many different and complex ways, and is likely to play a role in nutrient sensing, taste detection and regulation of appetite.

**Objective:**

To investigate to what extent oral and/or gut nutrient sensing of sweet impacts the entero-endocrine response after a mixed meal challenge test.

**Study design:**

A total of 20 participants (10 diabetes patients and 10 pre-diabetic controls) will be recruited. They will be matched for age and sex. The PhenFlex challenge, a mixed meal tolerance test developed by TNO will be used in combination with a preload to assess receive a mixed meal challenge in combination with oral or gastrointestinal priming with a sweetener or water, to determine the relation between nutrient sensing in the mouth and gut and the metabolic response of the gut. Participants will visit the research centre in Hoorn three times due to three different priming protocols, to achieve priming of the mouth, of the gut, and for a control measurement. Participants will first be offered a preload: in a random order, they will either rinse their mouth with a sweetened solution (oral priming), drink a sweetened solution (oral and gastrointestinal priming), or drink water (control). After 20 minutes, participants will receive a mixed meal challenge. The 20 minute interval was chosen based on previously conducted experiments testing preloads. Blood will be drawn once prior to priming (t=-20) and six times during the PhenFlex challenge test (t=0, 15,30, 60, 120 and 240), and analysed for the entero-endocrine response. In figure 1 a timeline to show sequence and timing of blood sampling is presented.


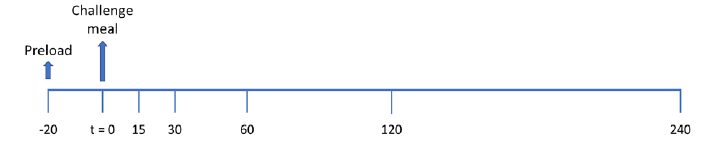


*Figure 1: Timeline of preload and challenge meal administration and blood sampling. The numbers indicate the time (in minutes) when blood samples are collected.*

In figure 2 a flowchart with the study procedures is presented.


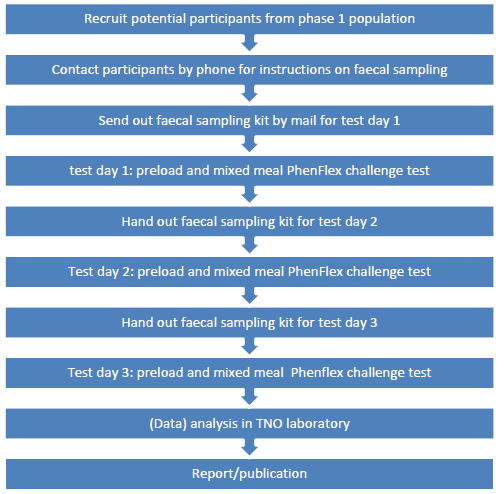


*Figure 2: Flowchart of study*

A computerized random number generator will be used to assign participants to different orders of the three test days.

**Study population:**

Participants are recruited from two study populations, DIRECT-1 and DIRECT-2, that were established for an observational EU study to establish biomarkers for Hba1c deterioration and was approved by the METC (NL40099.029.12). Participants were recruited in 2013. Both populations include white European men and women, 29-74 years of age, and able to speak, write and understand Dutch. DIRECT-1 consists of individuals at high risk of glycaemic deterioration, but without diabetes diagnosis; DIRECT-2 consists of individuals diagnosed with type 2 diabetes. The DIRECT-1 population in our locations consists of 480 men and women. The DIRECT-2 population in Hoorn consists of 110 men and women with type 2 diabetes. People are accustomed to participating in research, therefore we expect that the likelihood of obtaining adequate numbers of participants is high.

Inclusion criteria:

In order to be eligible to participate in this study, a participant must meet all of the following criteria:

- Able to speak, write and understand Dutch
- Voluntary participation
- Provided written informed consent
- Willing to comply with the study procedures of phase 1 and phase 2
- Appropriate veins and circulation for blood sampling
- Willing to accept use of all nameless data, including publication(s), and the confidential use and storage of all data for at least 15 years
- Willing to accept the disclosures of the financial benefit of participation in the study to the authorities concerned

Post-menopausal women will be preferably selected (when available). Similarly, age-matched men will be selected.

Exclusion criteria:

A potential participant who meets any of the following criteria will be excluded from participation in this study:

- Any significant medical reason for exclusion as determined by the investigator
- Having a history of medical or surgical (gastrointestinal) events that may significantly affect the study outcome
- Smoking
- Other medication for diabetes than oral medication (i.e. insulin)
- Recent antibiotic medication (in the last 3 months)
- Alcohol consumption >21 units/week
- Unable to give written informed consent
- Not willing to give up blood donation during the study
- Not having a general practitioner
- Not willing to accept information transfer concerning participation in the study, or information regarding his health, like laboratory results, findings at anamnesis or physical examination and eventual adverse events to and from his general practitioner

Withdrawal of participant:

Participants can leave the study at any time for any reason if they wish to do so, without any consequences. The investigator can decide to withdraw a participant from the study for urgent medical reasons. The reason for withdrawal will be registered. However, participants are free to stop with participation without mentioning the reason. Withdrawn participants cannot be replaced.

**Intervention:**

Steviol glycosides are administered to both groups as priming load before the mixed meal challenge.

**Study parameters/endpoints:**

Differences between control and diabetes patients in:

- Metabolic response to sweet by the gut
- The ways in which metabolic response and microbiome relate to each other

**Nature and extent of the burden and risks associated with participation, benefit and group relatedness:**

We do not foresee specific risks for the participants in the study. Participants will be asked to visit the research centre four times. Anthropometric measures will be taken once using non-invasive procedures. Three times blood will be collected, and the remaining samples will be obtained through non-invasive procedures (faeces collection and buccal/tongue swab).

**Investigational product**

During the three test days, steviol glycosides are used in combination with the mixed meal PhenFlex challenge to assess oral and gut nutrient sensing, by either a mouth rinse or swallowing of the steviolglycoside drink in comparison to a water control. The steviolglycoside drink will consist of 100 ml water sweetened with a pure steviolglycoside solution solution (Greensweet Stevia) to achieve a sweetness level similar to that present in soft drinks such as coca cola.

Steviol glycosides are natural constituents of the leaves of Stevia rebaudiana (Bertoni), a plant native to parts of South America and commonly known as Stevia. They are non-nutritive sweeteners and are reported to be 200 to 400 times sweeter than table sugar. Steviol glycosides are generally recognized as safe and permitted for use in food by the US Food and Drug Administration. Furthermore, EU Regulation 1131/2011, which came into force on 2 December 2011, permits steviol glycosides to be used in certain foods at permitted maximum levels (expressed as steviol equivalents). The EFSA Panel set an Acceptable Daily Intake (ADI) of 4 mg per kg body weight per day for steviol glycosides, a level consistent with that already established by the Joint FAO/WHO Expert Committee on Food Additives (JECFA).

**Non-investigational product**

Differences in sweet taste response of the gut and mouth to steviolglycosides will be tested by measuring the response to a challenge test in the form of a liquid mixed-meal (400ml) after an overnight fast, the so-called PhenFlex Challenge. The challenge meal will consist of 320ml tap water, 60g palm olein, 83.5g dextrose, 20g Protifar (protein supplement) and 0.5g artificial aroma (total energy content 950kCal). The mixed meal and sweetener solutions will be prepared under clean and standard conditions according to the basic rules of HACCP. In this way preparation of the product is standardized and registered, enabling review of the process if necessary. The participants will be asked to either rinse their mouth with the steviolglycoside drink, or to swallow the drink, or to swallow a blank (water) followed (after 20 min) by the challenge test drink, which has to be consumed within 5 minutes. Consumption will take place at the investigation site under supervision of the investigator and a study nurse.

**Study procedures**

Anthopometry:

Height, weight and the waist circumference will be measured. Blood pressure, systolic and diastolic, will be measured three times on the left arm, in sitting position after a five-minute resting period.

Swab for oral microbiome:

Two mucosal swab samples will be collected from the tongue dorsum. Tongue swabs will be collected using a sterile microbrush or Copan eNAT swab by applying 4 strokes over the most posterior or anterior part of the tongue dorsum. After the sample is taken, the tip of the microbrush/swab will be placed into a labelled Eppendorf vial with 250ul RNAProtect solution and clipped off. The sample will be frozen during the visit and transported to the clinical research unit. Upon arrival at the clinical research unit, the research personnel will immediately store the sample at -80degrees until molecular analyses at the TNO laboratory.

Faecal sample:

Participants will receive a fecal collection kit by post containing paper feces collection band (Fe-Col Alpha Laboratories), sample tubes (cylindrical containers with a spatula in the lid), a padded envelope and gloves. They will be instructed on how to use the feces collection band and tube both in writing and over the phone. The tube needs to be stored in the refrigerator at +- 4 degrees Celsius. Times of collection will be noted. These fecal samples will then be collected by the investigator at the time of the visit to the research centre. Fecal samples can be no more than 24 hours old. If the subject was unable to produce the sample within the set time frame, they will be instructed to try at a later time and send the sample to the research centre by post. At test day 2 and 3 participants will receive a new sample tube for sampling before the next test day. After arrival at the clinical research unit, the research personnel will immediately stir the tip of the spatula in a labeled Eppendorf vial with 400ul RNAProtect solution to release the faecal sample into the RNAProtect solution and store the sample at -80 degrees Celsius until 16S-rRNA metagenomics sequencing of microbial content by the TNO laboratory.

**Preload and mixed meal tolerance test**

The aim of this test is to determine to what extent oral and gut nutrient sensing affect the metabolic response of the gut in prediabetic and diabetic participants. To this aim, the PhenFlex challenge a mixed meal tolerance test developed by TNO, is used. For each participant, the response to the PhenFlex challenge in combination with a preload is assessed. The preload consists either of water (control) or of a solution of stteviolglycosides (SGs), to achieve priming of the mouth (by rinsing) or of the gut (by swallowing).

Due to three different priming protocols, the test is performed three times for each participant, on three separate days. Testing is done in the morning, and it lasts approximately 4.5 hours. Participants should arrive at the unit after an overnight fast i.e. no more eating and drinking is allowed form 22:00 the night before the test; some water is allowed). Furthermore, physical exercise should be avoided during the 24hours preceding the test.

The participant is offered a preload at time point t=-20. The preload should be consumed within 5 minutes. On the three different test days, the preload is one of the following:

- Water intake: the participant is asked to swallow 100ml of water
- SGs solution intake: the participant is asked to swallow 100ml of SGs solution
- SGs solution rinse: the participant is asked to rinse his mouth using 100ml of SGs solution, without swallowing it. The solution is offered 5 times, 20ml at the time, and each time the participant should swish it around in his/her mouth for 15 seconds, before expectorating. The participants can ten wait a few seconds before taking the next sip.

After 20 minutes from time point t=-20, the PhenFlex test meal is offered to the participant. The challenge drink should be consumed within 5 minutes. The order of the test days in which the participants are offered the different preloads is random.

**Blood collection**

During the preload and mixed meal tolerance test, blood will be collected seven times from the participants. The first blood sampling occurs at time point t=-20, just before offering the preload to the participant. The blood is collected again after 20 minutes, at time point t=0, just before offering the Phen Flex challenge drink to the participant. The blood is then collected 15, 30, 60, 120 and 240 minutes after the PhenFlex challenge. Total blood volume drawn during the study will be 240ml; up to 80ml per vist. Blood samples will be processed for plasma for storage, leptin, GLP1, GLP2, GIP, CCK and PYY analyses, and routine measurmeents glucose and insulin. The analyses will take place at TNO facilities (Zeist).

**Statistics**

Population characterstics will be shown as means and standard deviations, or in case of skewed distribution as median and interquartile ranges. Descriptive statistics are compared between intervention and control group in order to see whether there are significant differences between the two groups. If significant differences are found, further analyses will be adjusted for these confounders.

Microbiome data will be summarized using Simpson and Shannon diversity indices for each of the participant groups. Principal Coordinates Analysis using Bray-Curtis distance will be applied to obtain an overview of all the microbiome samples. If any sample appears to be anomalous, composition for all samples will be plotted using stacked bar charts in order to assess their composition.

We will compare the effect of the water control, mouth rinse and swallowing of the sweetener in repeated measurements. A two-tailed value of p<0.05 will be considered significant.

The hypothesis is that the variation in the plasma/serum measurements, either baseline or postprandial, will have equal means across the study protocols (water/mouth rinse/swallowing). Variation between the three test protocols will be analyzed by generalized linear model with the logit link functions using ‘treatment’ as a fixed effect and ‘participant’ as random factor. For the measurement’s obtained each of the sampling time points will be tested as if being independent. If any overall statistical differences between the three test protocols is found, post-hoc tests will be used to assess the origin of these differences.
